# Loss-of-function mutation in inositol monophosphatase 1 (IMPA1) results in abnormal synchrony in resting-state EEG

**DOI:** 10.1186/s13023-018-0977-1

**Published:** 2019-01-07

**Authors:** Christopher P. Walker, Andre L. S. Pessoa, Thalita Figueiredo, Megan Rafferty, Uirá S. Melo, Paulo R. Nóbrega, Nicholas Murphy, Fernando Kok, Mayana Zatz, Silvana Santos, Raymond Y. Cho

**Affiliations:** 1 Graduate School of Biomedical Sciences, University of Texas Health Science Center at Houston, Houston, TX USA; 2 Hospital Infantil Albert Sabin, Fortaleza, Brazil; 3 Universidade Estadual do Ceará-UECE, Fortaleza, Brazil; 4 Human Genome and Stem Cell Research Center, Department of Genetics and Evolutionary Biology, Instituto de Biociências, Universidade de São Paulo (USP), São Paulo, SP 05508-090 Brazil; 5 Department of Psychiatry, Baylor College of Medicine, Houston, TX USA; 6 Federal University of Ceará, Fortaleza, Brazil; 7 Department of Biology, State University of Paraíba (UEPB), Campina Grande, PB Brazil

**Keywords:** Inosotol monophosphatase, IMPA1, Electroencephalography, EEG, Oscillations

## Abstract

**Background:**

Dysregulation of the inositol cycle is implicated in a wide variety of human diseases, including developmental defects and neurological diseases. A homozygous frameshift mutation in *IMPA1*, coding for the enzyme inositol monophosphatase 1 (IMPase), has recently been associated with severe intellectual disability (ID) in a geographically isolated consanguineous family in Northeastern Brazil (Figueredo et al., 2016). However, the neurophysiologic mechanisms that mediate the *IMPA1* mutation and associated ID phenotype have not been characterized. To this end, resting EEG (eyes-open and eyes-closed) was collected from the Figueredo et al. pedigree. Quantitative EEG measures, including mean power, dominant frequency and dominant frequency variability, were investigated for allelic associations using multivariate family-based association test using generalized estimating equations.

**Results:**

We found that the *IMPA1* mutation was associated with relative decreases in frontal theta band power as well as altered alpha-band variability with no regional specificity during the eyes-open condition. For the eyes-closed condition, there was altered dominant theta frequency variability in the central and parietal regions.

**Conclusions:**

These findings represent the first human in vivo phenotypic assessment of brain function disturbances associated with a loss-of-function *IMPA1* mutation, and thus an important first step towards an understanding the pathophysiologic mechanisms of intellectual disability associated with the mutation that affects this critical metabolic pathway.

## Background

Dysregulation of the inositol cycle is implicated in a wide variety of human diseases including developmental defects, cancer, diabetes, and neurological diseases. A homozygous frameshift mutation in the gene coding for the enzyme inositol monophosphatase 1 (*IMPA1*) has recently been associated with severe intellectual disability (ID) in a geographically isolated consanguineous family in Northeastern Brazil [1]. Similar findings of ID have been found in another consanguineous cohort in Pakistan associated with an *IMPA1* mutation [2]. Preliminary case study MRI findings in a single subject from the Brazilian cohort revealed no structural abnormalities and no reduction of myo-inositol in the basal ganglia (consistent with similar findings in rodents) suggesting that neural disturbances may be more present and detectable at the circuit/systems level [1]. Therefore, we conducted a resting state electroencephalography (EEG) study with a subset of the Figueiredo et al. [1] cohort to test the hypothesis that the observed *IMPA1* loss of function mutation impairs neural circuits underlying normal brain functioning.

EEG allows for the non-invasive recording of coordinated activity across large populations of neurons with high temporal resolution. Scalp potentials measured by EEG reflect the summation of post-synaptic potentials along the apical dendrites of pyramidal neurons oriented perpendicular to the cortical surface [3]. EEG and other electrophysiology recording techniques yield measurements of cognitive and sensory networks in the form of quasi-stationary neural oscillations that offer an in vivo index of circuitry-level neurophysiologic function. More significantly, modern EEG equipment is highly mobile and can be easily transported to conduct novel field research in isolated regions.

Unfortunately, there have been no human or animal electrophysiologic studies to date investigating the effects of IMPA1 or IP3 accumulation, a downstream effect of IMPA1 inhibition. In mice, *Impa1* knockout is lethal during embryonic development if inositol is not supplemented in the mother’s diet [4]. Postnatally, clear behavioral effects are observed in homozygous *Impa1* mutant mice rescued by dietary myo-inositol, including hyperlocomotion and altered circadian rhythms [5]. This developmental lethality was notably absent in the Figueiredo et al. (2016) [1] cohort which, in the presence of the ID phenotype, suggests the presence of imperfect redundancies or compensatory mechanisms in the inositol cycle pathways that alter neurophysiologic function.

Due to the absence of prior human and animal neurophysiology studies of *IMPA1/Impa1* mutation, we could not use such literature to develp an *a priori* hypothesis of an electrophyiological biomarker of *IMPA1* mutation. Therefore, we drew upon the closest pharmacological model of IMPA1 inhibition with a rich neurophysiological literature: lithium. Prior rodent work suggests lithium acts through a combination of Impa1 inhibition and reduced *Smit1* mRNA expression [6]. In particular, lithium inhibition of inositol monophosphatase was shown in cell culture to increase IP1 (inositol phosphate-1) concentrations which in turn mediated the rate of phosphoinositide synthesis [7]. We reasoned that a loss-of-function mutation of *IMPA1* may result in a similar disruption of the inositol metabolic cycle as lithium administration, and as such, we may use human EEG studies of lithium administration to generate testable hypotheses. In human EEG studies, lithium has been found to enhance early sensory potentials, increase low frequency activity in resting state EEG, and increase event-related beta oscillations [8, 9]. While it is unclear the degree to which IMPA1 related mechanisms specifically influence lithium-induced EEG effects, we believe previous research into the effects of lithium on human EEG may offer a useful framework for guiding our predictions.

To that end, we hypothesized that homozygous carriers of a loss of function *IMPA1* mutation would resemble healthy controls under chronic lithium administration and show elevated low frequency activity (i.e., delta, theta, and alpha oscillations) compared to carriers of the wild-type form of *IMPA1*. To test this hypothesis, we collected resting-state EEG recordings under eyes open and eyes closed conditions in the Figueiredo et al. (2016) [1] cohort and computed standard quantitative EEG measures of frequency band power and variability. We identified an electrophysiological phenotype that did not follow the predictions of increased low-frequency power, but rather identified increased low-frequency variability as a potential characteristic of the loss of function mutation identified in this cohort.

## Methods

### Participants

Thirty participants in the family identified in Figueiredo et al. (2016) [1] were recruited to participate in the current study. Of the 30, four participants were homozygous for the mutant *IMPA1* allele (c.489_493dupGGGCT) (hereafter called HOM, 4 female, 0 male). Nine participants carried only one copy (HET, 2 female, 7 male), and the remaining 17 carried the normal variant (WT, 11 female, 6 male). All HOM patients showed the intellectual disability phenotype. Several attempts were made to recruit additional HOM patients; however, several affected patients were notably irritable and unable to sit for the EEG. All participants were evaluated for neuropsychiatric co-morbidities through the Mini-International Neuropsychiatric Interview (MINI-6.0, Portuguese version). Eight participants were identified as exhibiting symptoms of psychosis (2 HOM, 3 HET, & 3 WT); however, we observed no statistical relationship between genotype and the presence of psychosis symptoms (Fisher’s Exact Tests, all ps > 0.50). Most participants were taking medications (antipsychotics: 1 HOM, 2 HET, 1 WT; benzodiazepines: 1 HOM, 1 HET, 3 WT; SSRIs: 1 WT; antihypertensives: 1 HOM, 4 HET, 5 WT; oral hypoglycemic: 1 HET; allopurinol: 1 WT). Additional demographic information is reported in Table 1.

**Table 1:** Subject demographics

IMPA1 Mutation	*n*	Female	Male	Psychosis	Age (SD)
HOM^+/+^	4	4	0	2	51.5 (5.3)
HET^+/−^	9	2	7	3	44.8 (12.6)
WT^−/−^	17	11	6	3	45.0 (12.4)

### Electroencephalographic recordings

Resting EEG data were collected in an air-conditioned room under eyes open and eyes closed conditions (2 min each). EEG data were collected from a 32 channel ActiCAP with active Ag/AgCl electrodes using a BrainAmpMR amplifier (Brain Products, Munich, Germany). Scalp locations were based on the International 10/20 system. Data were digitized at 5000 Hz with a 0.1 to 1000 Hz hardware band-pass filter. Ground and reference electrodes were placed at AFz and FCz respectively. Electrode impedances were maintained at or below 20 kΩ. For the final 11 participants (9 WT, 3 HET), the ActiCAP electrode impedance measurement failed, preventing accurate recording of impedances. Therefore, online EEG data were evaluated by two experts for overall quality. As an additional index of data integrity, an on-line running average of visual evoked potentials from a separate EEG task were reviewed during the recordings to determine that the EEG data collected were valid.

### EEG preprocessing and analysis

Data preprocessing was done offline using custom Matlab scripts (Mathworks, Natick, MA). First, Continuous EEG data were notch filtered from 59 to 61 Hz followed by a 0.2 to 150 Hz band-pass filter. Data were then segmented into 2500 ms epochs for artifact identification. Individual trials and channels were rejected based on statistical distance from channel and trial means and variances. Data which were deemed to be contaminated by artifact were removed from the data, the remaining clean data were submitted to independent components analysis (ICA) [10]. Briefly, ICA is a blind signal-source separation technique that decomposes mixed signals (e.g., scalp EEG) into unmixed ‘components’ based on spatial patterns of activity. Each ICA component acts as a spatial filter defining unique sources of activity by a weighted sum of EEG channels which can be subtracted from the raw data to remove modeled artifacts. Stereotyped artifacts such as blinks, eye movements, heartbeats, and muscle artifacts were visually inspected and removed by expert reviewers [11, 12]. Artifact free data were submitted for a second round of trial and channel evaluation. Individual channels/trials exceeding a 3 standard deviations of the sample mean of the data were removed from the final analysis.

To evaluate the influence of the *IMPA1* mutation on the spectral profile of resting EEG, two quantitative EEG (qEEG) measures were derived from the power spectrum densities (PSD) of clean EEG data. First, mean band power (MBP) was calculated using Welch’s method of windowed averaging over a time series [13]. Window sizes were set to half of the data segment length (i.e., 1250 ms) with no window overlap to avoid discontinuities across potentially non-adjacent epochs (final frequency resolution = 0.63 Hz). PSDs were converted to relative PSD by normalizing each subject’s estimate by the sum power over all frequencies up to 100 Hz. Band power was then defined as the mean power across 6 canonical frequency band: (1) Delta, δ < 4 Hz; (2) theta, θ = 4–8 Hz; (3) alpha, α = 9–14 Hz; (4) beta, β = 14–30 Hz; (5) low gamma, low γ = 31–55 Hz; (6) high gamma, high γ = 65–100 Hz. Lastly, we estimated oscillatory network stability by computing dominant frequency variability (DFV) over trials. In each 1250 ms window, the frequency exhibiting the maximum power was identified within frequency bands. DFV was defined as the mean of absolute deviation scores (i.e., $DFV=\frac{\sum \left(\left|X-\mu X\right|\right)}{n}$). Mean absolute deviation was used to reduce the potential influence of outlier trials on the variability estimation (i.e., compared to standard deviation). MBP and DFV scores were summarized by averaging estimates within 8 scalp regions (i.e., left frontal, middle frontal, right frontal, left temporal, central, right temporal, parietal, occipital). Matlab scripts used for this analysis can be found at http://github.com/cholab/IMPA1-EEG.

### Statistical analysis

To test for associations between the *IMPA1* genotype and the observed qEEG phenotypes, we employed a multivariate family-based associate test using generalized estimating equations (FBAT-GEE; FBAT-Toolkit v204, http://sites.google.com/view/fbat-web-page) [14]. Briefly, FBAT-GEE is an extension of a traditional FBAT that tests for associations between allelic presence and a given phenotype while conditioning the null hypothesis of the test statistic on the observed phenotype distribution. FBAT-GEE expands on the traditional FBAT statistic given by

1 $$ {\chi}^2=\frac{{\left(S-E(S)\right)}^2}{V_S} $$

where $S=\sum \limits_{i=1}^n{t}_i{x}_i$, the expected value, $E(S)=\sum \limits_{i=1}^n{t}_iE\left({x}_i|{p}_{i1},{p}_{i2}\right)$ …, and the variance ${V}_s=\sum \limits_{i=1}^n{t}_i^2 Var\left({x}_i|{p}_{i1},{p}_{i2}\right)$. By this metric, both quantitative (i.e., continuous) or categorical (i.e., bivariate) phenotype may be represented as t_i_ for the *i*th individual. The value of x_i_ codes for the hypothesized phenotype expression based on allele frequency for the marker of interest (i.e., dominant, additive, or recessive). Both E(S) and V_s_ set the normalization parameters to be conditional on the genotype expression in the parents.

The multivariate extension replaces S and E(S) with the m-dimensional vector $\overset{\sim }{S}$ defined as

2 $$ \overset{\sim }{S}=\sum \limits_{i=1}^n{t}_i\left({x}_i-E\left({x}_i|{p}_{i1},{p}_{i2}\right)\right) $$

where *m* is number of phenotypes being tested, and the variance is given by the *m* x *m* matrix defined as

3 $$ {V}_{\overset{\sim }{S}}= Var\left(\overset{\sim }{S}\right)=\sum \limits_{i=1}^n{t}_i{t}_i^t Var\left({x}_i|{p}_{i1},{p}_{i2}\right) $$

where ^t^ signifies a vector transpose. The final FBAT-GEE statistic is thus given by

4 $$ {\chi}_{FBAT- GEE}^2={\overset{\sim }{S}}^t\ {V}_{\overset{\sim }{S}}^{-1}\ \overset{\sim }{S} $$

which is asymptotically *χ*^2^-distributed with degrees of freedom equal to $k=\operatorname{rank}\left({V}_{\overset{\sim }{s}}\right)$. The statistical inference follows that a significant test statistic indicates the association between an allele loading and the observed phenotype is unlikely to occur by chance given the observed distribution of alleles in the parents. Since the intellectual disability associated with the *IMPA1* mutation has previously demonstrated a strongly recessive pattern, we elected to use a recessive FBAT model where *aa* carriers are coded as 1, and AA and Aa carriers are coded as 0.

The recessive FBAT-GEE model was employed based on the *a priori* assumption that the inheritance pattern would follow that of the more global phenotype of intellectual disability. However, since the narrower neurophysiologic phenotypes investigated here may show expression even with partial allelic loading, as an additional exploratory step, we repeated our FBAT-GEE analyses with the additive model (i.e., AA = 0, Aa = 1, and aa = 2) to identify potential phenotypes that are more sensitive to such partial loading.

Statistical analyses were conducted in the FBAT-GEE framework for each frequency band and qEEG measure with scalp region acting as the multivariate dimension. Univariate FBATs were run post-hoc within each region to determine if effects were global or regional. Owing to the small sample size, the uniqueness of the mutation identified in the study population, and the generally exploratory nature of our investigation, all tests were evaluated at α = 0.05, uncorrected. Therefore, we present the following analysis under the framework of hypothesis generation, and stress the need for confirmatory follow-up experiments.

## Results

Quantitative EEG measures including mean power and dominant frequency variability were extracted from resting EEG data of 30 participants (17 WT, 9 HET, 4 HOM). From this sample, 27 subjects (14 WT, 9 HET, 4 HOM) were from the eight families informative for the *IMPA1* mutation and therefore chosen for our analysis.

**Fig. 1:**
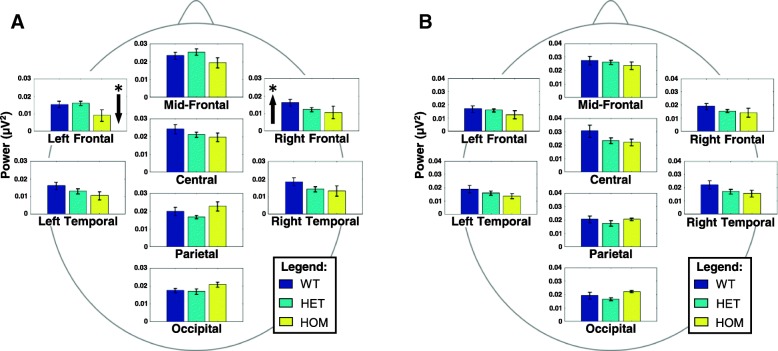
Mean (± SEM) theta-band power by scalp region and genotype for eyes open and eyes closed conditions. (BLUE = WT, TEAL = HET, YELLOW=HOM). Arrows indicate the direction of significant associations between theta-band power and specific IMPA1 alleles under the recessive FBAT model. Arrows on the left of a graph indicate an association with the wild-type allele within a specific region. Arrows on the right of the graph indicate an association with the mutant allele within a specific region. **a** In the eyes open condition, significantly lower left frontal theta power was associated with the mutant allele, and significantly greater right frontal theta power was associated with the wild-type allele. The right frontal pattern was also present under the additive FBAT model. **b** In the eyes closed condition, these differences were not observed. This lateral shift in theta-band power representations on the scalp suggests subtle anatomical or network-level differences may arise during development in the presence of the *IMPA1* mutation

**Fig. 2:**
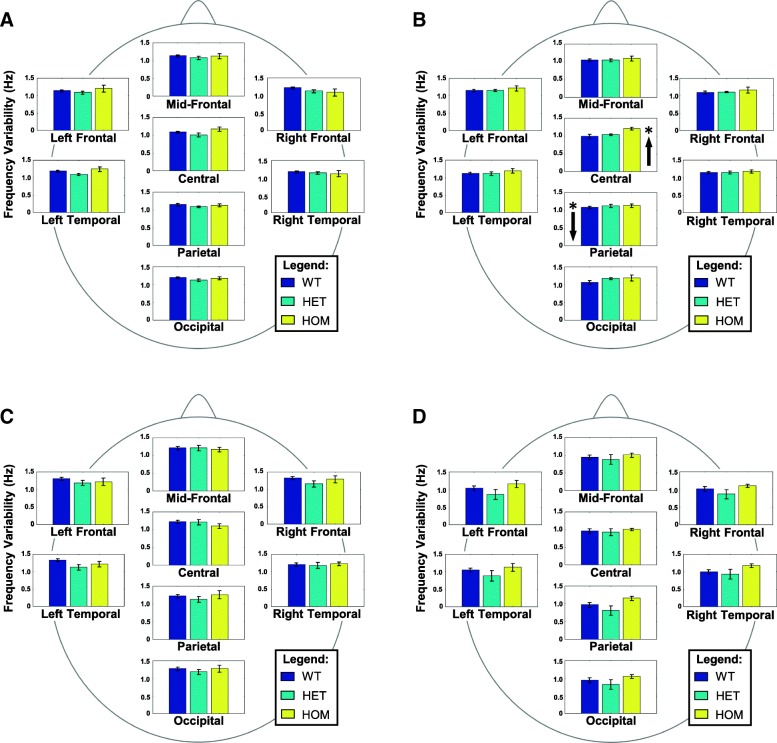
Mean (± SEM) dominant frequency variability by scalp region and phenotype. (BLUE = WT, TEAL = HET, YELLOW=HOM). Arrows indicate the direction of significant associations between theta-band power and specific IMPA1 alleles under the recessive FBAT model. Arrows on the left of a graph indicate an association with the wild-type allele within a specific region. Arrows on the right of the graph indicate an association with the mutant allele within a specific region. **a** No significant associations were observed for theta-band variability in the eyes open condition. **b** In the eyes closed condition, the mutant allele was associated with a significantly higher central theta variability, and the wild-type allele was associated with significantly lower parietal theta variability. These patterns supplement the power results suggesting a core theta-band oscillatory disturbance resulting from the *IMPA1* mutation. **c** Eyes open and **d** eyes closed alpha-band variability did not show any regional associations with specific alleles; however, multivariate-FBAT analyses found significant associations between the wild-type allele and alpha variability in the eyes open condition suggesting a more global phenomenon

Both quantitative EEG measures were natural log transformed prior to FBAT testing to better approximate a normal distribution. We performed FBAT analysis to evaluate the association between the *IMPA1* mutation and our quantitative phenotypes (i.e., MBP, and DFV). See Tables 2 and 3 for a summary of results. First, a multivariate FBAT-GEE was performed for each of the 6 frequency bands across all scalp regions; significant associations were found with θ-band power (χ^2^ = 18.451*, p* = .018) and dominant α-band variability (χ^2^ = 19.771, *p* = .011) for the eyes-open condition, and dominant θ-band variability (χ^2^ = 15.848, *p* = .045) for the eyes-closed condition. With these identified frequency bands, the 8 individual scalp regions were subsequently tested with univariate analysis. For the eyes-open condition, lower than expected θ power over the left frontal scalp region was significantly associated (*Z* = − 2.211, *p* = .027) with the mutated allele, while higher than expected θ power over the right frontal scalp region was significantly associated (*Z* = 2.248, *p* = .025) with the wild-type allele (see Fig. 1). For the eyes-closed condition, dominant θ variability over the central scalp region was significantly associated (*Z* = 2.411, *p* = .016) with the mutated allele, while dominant θ variability over the parietal scalp region was significantly associated (*Z* = − 2.329, *p* = .020) with the wild-type allele (see Fig. 2). Univariate FBATs showed no significant associations for alpha, indicating a general effect across the scalp for dominant α-band variability.

**Table 2:** FBAT-GEE for eyes-open condition

	Allele	Freq.	Df	Recessive		Additive	
				χ^2^	*P*	χ^2^	*P*
Mean band power							
Delta	1	0.836	8	9.220	0.3241	–	–
	2	0.164	8	11.235	0.1888	11.426	0.1787
Theta	1	0.836	8	18.451	0.0181*	–	–
	2	0.164	8	11.220	0.1895	16.388	0.0372*
Alpha	1	0.836	8	13.891	0.0847	–	–
	2	0.164	8	8.957	0.3459	14.097	0.0793
Beta	1	0.836	8	11.385	0.1808	–	–
	2	0.164	8	11.364	0.1819	10.728	0.2176
Gamma 1	1	0.836	8	7.371	0.4972	–	–
	2	0.164	8	10.567	0.2275	9.196	0.3261
Gamma 2	1	0.836	8	8.811	0.3585	–	–
	2	0.164	8	11.431	0.1785	12.598	0.1265
Dominant frequency variability							
Delta	1	0.836	8	5.703	0.6805	–	–
	2	0.164	8	11.948	0.1535	10.175	0.2530
Theta	1	0.836	8	11.101	0.1961	–	–
	2	0.164	8	9.636	0.2915	12.961	0.1132
Alpha	1	0.836	8	19.771	0.0112*	–	–
	2	0.164	8	5.415	0.7124	11.427	0.1786
Beta	1	0.836	8	5.137	0.7428	–	–
	2	0.164	8	5.788	0.6710	6.108	0.6351
Gamma 1	1	0.836	8	8.782	0.3610	–	–
	2	0.164	8	5.718	0.6788	8.791	0.3603
Gamma 2	1	0.836	8	12.742	0.1210	–	–
	2	0.164	8	1.749	0.9878	7.530	0.4806

**Table 3:** FBAT-GEE for eyes-closed condition

	Allele	Freq.	Df	Recessive		Additive	
				χ^2^	*P*	χ^2^	*P*
Mean band power							
Delta	1	0.836	8	7.534	0.4802	–	–
	2	0.164	8	5.859	0.6630	8.326	0.4023
Theta	1	0.836	8	15.366	0.0524	–	–
	2	0.164	8	9.769	0.2816	14.265	0.0751
Alpha	1	0.836	8	10.257	0.2474	–	–
	2	0.164	8	10.917	0.2065	14.115	0.0788
Beta	1	0.836	8	11.398	0.1802	–	–
	2	0.164	8	6.49	0.5925	10.214	0.2503
Gamma 1	1	0.836	8	8.878	0.3527	–	–
	2	0.164	8	8.486	0.3875	11.915	0.1550
Gamma 2	1	0.836	8	5.378	0.7165	–	–
	2	0.164	8	7.796	0.4537	7.854	0.4478
Dominant frequency variability							
Delta	1	0.836	8	9.147	0.3301	–	–
	2	0.164	8	8.009	0.4326	12.291	0.1387
Theta	1	0.836	8	15.848	0.0446*	–	–
	2	0.164	8	10.01	0.2644	13.985	0.0822
Alpha	1	0.836	8	15.264	0.0542	–	–
	2	0.164	8	8.499	0.3863	14.543	0.0687
Beta	1	0.836	8	8.618	0.3756	–	–
	2	0.164	8	9.717	0.2855	12.228	0.1413
Gamma 1	1	0.836	8	9.717	0.2855	–	–
	2	0.164	8	8.698	0.3684	12.144	0.1449
Gamma 2	1	0.836	8	6.865	0.5513	–	–
	2	0.164	8	9.659	0.2898	10.571	0.2272

As noted above, we conducted FBAT-GEE analyses employing the additive model as an exploratory analysis. This showed a significant association (χ^2^ = 16.388, *p* = .037) between θ-band power and the mutated allele for the eyes-open condition. Univariate analysis showed lower than expected θ power over the right frontal scalp region was found to be significantly associated (*Z* = − 2.542, *p* = .011).

## Discussion

In the current study, we conducted a resting EEG study to investigate the electrophysiologic phenotype associated with the *IMPA1* mutation in a geographically isolated, consanguineous cohort in Brazil [1]. Our primary findings with the *IMPA1* mutation being associated with relative decreases in left and right frontal θ power and altered α-band variability with no identifiable regional specificity during the eyes-open condition. For the eyes-closed condition, there was altered dominant θ frequency variability in the central and parietal regions. These findings differ from the elevated low-frequency activity that would be hypothesized based on such findings in healthy individuals under lithium administration. However, it is not overly surprising that our findings may depart from these simple predictions based on acute lithium administration, given the complexity of the inositol metabolic pathway, the comparative timeframes involved, and specific population being investigated. Regardless, the patterns identified in this analysis will need to be replicated in future studies to establish the generalizability of these effect to other carriers of the *IMPA1* mutation and IMPA1 function more generally.

The *IMPA1* mutation in the cohort of the current study was associated with severe ID and disruptive behavior. Most homozygous individuals found it very challenging or were unable to complete simple computer or neuropsychological tests of cognitive and intellectual functioning (and a number of identified individuals could not participate at all due to the severity of their impairment and debilitation). Resting-state EEG placed minimal burden on participants and as such was an ideal approach for probing neural phenotypic expression of the *IMPA1* mutation in this population. While no cognitive process is actively engaged through explicit task performance, such intrinsic resting state activity is nevertheless thought to reflect the activity and functional integrity of networks that support various cognitive and perceptual processes [15, 16] and segregates into distinct, separable frequency bands [17].

In the current study, two prominent rhythms in resting EEG, θ-band and α-band activity, showed variation with the *IMPA1* mutation. Theta oscillations have been associated with cognitive control functions such as error monitoring [18] and working memory [19, 20] including the modulation of local gamma activity [21] and mediating cortical interactions with the hippocampus [22]. In our cohort, we found evidence of reduced frontal theta power, in association with intellectual disability. While formal assessment of working memory and cognitive control was not possible due to the level of impairment in individuals homozygous for *IMPA1* mutation, it seems highly likely that the disturbances in theta were associated with severe impairments in both these cognitive processes. Interestingly, both these cognitive processes are highly correlated with IQ [23–26] and thus impairments in these fundamental cognitive functions may underlie the significant intellectual disability present in our cohort. Furthermore, altered θ-band and α-band oscillations have been observed in other disorders associated with intellectual disability including fragile X syndrome (FXS) [27, 28] and attention-deficit hyperactivity disorder (ADHD) [29, 30]. The precise alterations differ between specific disorders, but suggest θ-band and α-band oscillations represent a potential convergent mechanism underlying intellectual disability across etiologies. Further research will be necessary to evaluate the usefulness of θ/α oscillations as a generalizable biomarker of ID.

### Putative mechanisms of alpha and theta oscillations

Alpha-band oscillations are thought to underlie a different putative function. By and large, alpha oscillations are considered to reflect cortical inhibitory processes which can serve to suppress or gate information transfer in the brain (i.e., the inhibition-timing hypothesis) [31, 32]. In the context of cognitive tasks, alpha oscillations are considered to reflect a top-down control mechanism to inhibit task-irrelevant or task-distracting information [33]. By contrast, decreases in alpha activity are often observed in task relevant brain regions [34–38]. In the sensory domain, the phase of ongoing alpha oscillations have been shown to influence bottom-up perceptual fidelity [39, 40] which similar to theta oscillations may help bind information carried and maintained in high frequency, gamma-band oscillations [41]. This balance between suppression and gating functions are thought to be driven by thalamocortical neurons via muscarinic acetylcholine (mAChR) and metabotropic glutamate type-1 receptors (mGluR1) [42, 43]. Notably, mAChRs are Gq-protein coupled receptors known to upregulate inositol triphosphate (IP3) through activation of phospholipase C [44]. Given the role of IMPA1 in modulating the production of IP3, our finding of a significant multivariate FBAT association between the wild-type allele and alpha-band variability suggests that intact IMPA1 functioning may support the flexible coordination of thalamocortical alpha rhythms, which in the absence of a sufficient supply of IP3, results in a decrease in alpha-band variability for homozygous carriers of the *IMPA1* mutation.

The theta disturbances in the current study manifested in both the power and dominant frequency variability of theta in association with the *IMPA1* mutation. The power of any oscillatory rhythm is dependent on a number of factors including the numbers of pyramidal neurons and synapses giving rise to the rhythm and how synchronous their activations are. It is not yet known the degree to which *IMPA1* mutation may be associated with decreased neuronal number or disturbances in synaptic morphology or function. However, *Impa1* knockout has been found to increase autophagy [6] which, in a non-physiologic context, may be deleterious to synaptic function or morphology and thus diminish EEG theta power. How the *IMPA1* mutation may affect the dominant frequency is also unclear, but potential mechanisms are suggested by studies of calbindin (CB), which can activate IMPase [45–47]. CB suppression in hippocampal excitatory neurons has been associated with memory impairments and is thought to be mediated by downstream effects on IMPase [47]. The firing of hippocampal CB positive pyramidal cells is strongly locked to theta rhythms in the context of spatial navigation and memory [48, 49]. It is not clear that such findings in the hippocampus would have relevance to the cortical theta rhythms that are detectable by EEG as in the current study. However, cortical theta rhythms arise from the coordination of CB positive basket interneurons and pyramidal cells which, in turn, may have monosynaptic input from the ventral hippocampus and thus be modulated by the strong theta rhythms generated there [50]. Thus, if the kinds of CB suppression effects as observed in the hippocampus are mediated by impact on IMPase function, the *IMPA1* mutation could lead to disturbances in the coordination of network activity that manifest as instabilities in the dominant frequency of cortical theta rhythms.

Our findings of impaired cortical theta rhythms run counter to the predictions that would be made taking lithium as a model of *IMPA1* mutation. Administration of lithium leads to reductions in IMPA1 activity and enhancements in low-frequency EEG rhythms [8]. However, lithium also results in reduced *Smit1* mRNA expression [6] and thus it is not clear what may lead to the observed enhancements in theta. Further, even if findings of increased theta with lithium were attributable to decreased IMPA1 function, the impact of an *IMPA1* mutation may be fundamentally different from lithium administration. In the case of an *IMPA1* mutation, the protracted course of effects could start as early as in utero and impact a neurodevelopment course that may also involve compensatory mechanisms in inositol metabolism and associated pathways that together result in neurophysiologic outcomes altogether different from much shorter time scale lithium administration in adults.

### Strengths and limitations

Our study had a number of strengths, notably including the uniqueness of the study sample and the successful collection of electrophysiologic measures in a remote, rural setting. The mobility of our EEG equipment facilitated access to a geographically isolated sample which allowed the study team to bring the lab to the participants. We found that the homozygous carriers of the *IMPA1* allele were too impaired to accurately assess IQ or perform cognitive behavioral tasks during EEG. As such, the data was collected under passive resting state conditions, avoiding reliance on subject motivation and attention. Thus, the present findings are not confounded by differences in attentional engagement as may be the case in cognitive or sensory tasks. The present data supports the feasibility of data collection and meaningful analysis using a resting state paradigm in this population.

There are some limitations that should be considered when interpreting the results. While it is the first study of its kind to investigate the *IMPA1* mutation employing human electrophysiology, the sample size was limited. This was to large extent unavoidable due to a number of factors including the geographically isolated nature of the population, the inability of a number of homozygous *IMPA1* mutation candidates to participate in the study because of the severity of their impairment, and the lack of on-site lab facilities and expertise to conduct longer-term data collection. We conducted a high-powered multivariate statistical analysis of the data to capitalize on the familial pedigree structure of our sample, but were still limited by the sample size which was underpowered relative to the conservative corrections for our post-hoc multiple comparisons. However, given the rarity of the sample, we elected to present the findings of the current study with the understanding that they require replication in additional cohorts. On a related note, despite the much higher proportion of psychosis exhibited in individuals who were homozygous or heterozygous for the mutant allele, the sample size limited a more definitive examination of the phenotypic relationship between intellectual disability and psychosis in the context of the *IMPA1* mutation. Such associations between psychiatric symptoms and cognitive impairments are common in neuropsychiatric disorders such as schizophrenia, but the precise nature of the relationship will require much larger samples, perhaps primarily comparing heterozygous to wildtype groups given the severe cognitive impairment manifesting in individuals homozygous for the mutant allele. Finally, future investigations could also attempt higher-density EEG recordings to facilitate anatomic source analyses of the resting state networks.

## Conclusion

We conducted the first electrophysiologic phenotyping human study of the *IMPA1* mutation in a geographically isolated, consanguineous cohort. Our findings of disturbances in frontal theta and more global alpha band disturbances raises the possibility of perturbations in certain cellular subtypes and aspects of the inositol metabolic pathways. More mechanistically definitive studies will require preclinical models with controlled manipulation of the *IMPA1* expression and electrophysiological recordings. Based on these important preliminary findings, now we are expanding the studies to patient-derived neuronal cell lines in order to clearly elucidate the mechanisms by which impairment of IMPA1 can alter important signaling pathways that may lead to development of intellectual disability and to correlate with in vivo measures of brain activity and cognitive capacity to better understand the pathway from genetic variants to behavior. To our knowledge, however, this current study is the first human in vivo phenotypic assessment of brain function disturbances associated with the *IMPA1* mutation, thus representing an important first step towards understanding the pathophysiology of intellectual disability associated with the mutation that affects this critical metabolic pathway.

## Abbreviations

CB: Calbindin
DFV: Dominant frequency variability
EEG: Electroencephalography
FBAT-GEE: Family-based association test with generalized estimating equations
HET: Heterozygous
HOM: Homozygous
ICA: Independent components analysis
ID: Intellectual disability
IMPA1: Inositol monophosphatase 1
IP3: Inositol triphosphate
IQ: Intelligence quotient
mAChR: Muscarinic acetylcholine receptor
MBP: Mean band power
mGluR1: Metabotropic glutamate type-1 receptor
mRNA: Messenger ribonucleic acid
SEM: Standard error of the mean
SMIT1: Sodium-myoinositol cotransporter 1
WT: Wild-type
